# Comparing physical activity prescription with verbal advice for general practice patients with cardiovascular risk factors: results from the PEPPER randomised controlled trial

**DOI:** 10.1186/s12889-023-16302-6

**Published:** 2023-07-20

**Authors:** William Bellanger, Matthieu Peurois, Laurent Connan, Nastassia Navasiolava, David Missud, Thibaut Py, Cyril Bègue

**Affiliations:** 1 Department of General Practice, University of Angers, 49000 Angers, France; 2 University of Angers, Univ Rennes, EHESP, Inserm, IRSET-ESTER,, SFR ICAT, 49000 Angers, France; 3 University of Angers, CHU Angers, CRC, INSERM, CNRS, MITOVASC, Equipe CarMe, SFR ICAT, 49000 Angers, France

**Keywords:** Physical activity prescription, Cardiovascular risk, Pedometer, Primary care

## Abstract

**Background:**

Regular physical activity improves health and quality of life for people with cardiovascular risk factors. However, few studies have demonstrated the applicability of strategies in health care to promote physical activity.

**Objective:**

To evaluate if a written physical activity prescription combined with pedometer increases physical activity over one year compared with verbal advice in patients with cardiovascular disease risk in primary care.

**Methods:**

The randomised-controlled, interventional, 12-month PEPPER study recruited patients aged 35 to 74 years, having quarterly followed-ups for hypertension, dyslipidaemia, or diabetes, and judged insufficiently active. Seventeen practices randomised patients into either the experimental group, who received a written, personalised prescription for daily step numbers, pedometer and logbook, or control group, who received verbal advice to do at least 15 min of rapid walking or equivalent daily. The primary outcome was the change in total weekly energy expenditure measured using an accelerometer at 3 months. The secondary outcomes were changes in step count, physical activity levels, quality of life, perceived obstacles to physical activity, and biomedical indicators at 3 and 12 months.

**Results:**

One hundred and twenty-one participants were randomised. Although, weekly energy expenditure did not differ between the prescription and verbal instruction group, the estimated time spent doing moderate-intensity activity was significantly higher in the prescription group than the verbal group by an average of four minutes/week (*p* = 0.018)(95% CI [0.7 – 7.4]) reaching 48 min after 12 months (95% CI: 8 – 89). Similarly, this was associated with a clinically, higher average step number of 5256 steps/week increase over a year (95% CI: 660 – 9852). Among the most sedentary subgroup, walking less than 5000 steps/day at baseline, an 8868 steps/week (95% CI [2988 – 14700]) increase was observed in the prescription group.

**Conclusion:**

Prescribing physical activity did not significantly modify total weekly energy expenditure, but slightly increased moderate-intensity activity duration and step counts, particularly among the most sedentary participants. Prescribing personalised physical activity goals encourages sedentary patients to engage in physical activity.

**Trial registration:**

The PEPPER trial is registered in the US National Institutes of Health Clinical Trials Registry under number NCT02317003 (15/12/2014).

**Supplementary Information:**

The online version contains supplementary material available at 10.1186/s12889-023-16302-6.

## Background

Physical activity is among numerous possible therapeutic and preventive strategies to reduce the risk of cardiovascular disease and improve patient health indicators, quality and life expectancy [1–7].

Various interventional strategies to promote physical activity and reduce cardiovascular disease risks have been evaluated, most of which were based on verbal advice and recommendations [8–13]. However, the most effective strategies are often complex, involving regular follow-up telephone calls or meetings with support from nurses or other health professionals [14]. Unfortunately, these strategies are difficultly transposable to France where general practitioners (GP) have limited healthcare professional support networks. Pedometers offer a simpler strategy. However, despite pedometers showing short-term efficacy, the British National Institute for Clinical Excellence highlighted the current insufficient evidence to generalise their use in primary care [14]. Furthermore, few studies have evaluated the long-term motivational effect of pedometer use beyond 6 months.

Additionally, written prescriptions for physical activity have been shown to be a feasible interventional strategy in ambulatory care with measurable objectives [15]. Advantages of this strategy include the ability to evaluate self-determined objectives [16], implement graded targets for patients [8, 17], address obstacles to changing lifestyle habits [18], self-assess behaviour and performance [16], monitor pedometer use [19–22], perform regular follow-ups and provide encouragement [17].

For these reasons, a simple strategy for GPs in France to improve physical activity among their patients with cardiovascular risk factors could be a written prescription for physical activity, tracked with a pedometer and logbook.

The objective of this study is to evaluate whether written physical activity prescription combined with a pedometer would increase physical activity more than verbal advice over one year in patients with cardiovascular risk factors in primary care.

## Materials and methods

### Study design and participants

The PEPPER study (Prescription d’Exercice Physique avec Podomètre) was a prospective, comparative, open, multicentre, randomised-controlled trial with a parallel design. The study protocol was published in 2019 [23]. The study was conducted between March 2015 and April 2019, involved seventeen general practices in the Pays-de-la-Loire region of France and recruited adult patients aged between 35 and 74 years who were being followed-up every three months for hypertension, hypercholesterolaemia, or non-insulin dependent (type 2) diabetes, and whose level of activity was judged insufficient.

Potential participants were included if they answered ‘no’ to both of the following questions asked by their GP:

“Do you practice any sport, including cycling, for an hour or more every week?”

“Does your work involve physical activity?”.

Patients were not considered for enrolment if they had a health condition that contraindicated moderate physical activity or were unable to walk without human or material assistance. Patients were excluded if they had a health condition including a psychiatric or cognitive impairment affecting their judgement meaning they were unable to understand the GP’s advice, had another serious chronic pathology (such as severe coronary disease or heart failure), or did not speak French or refused to participate were excluded.

Once enrolled, GPs informed the participants about the study and all participants provided signed informed consent before any study interventions took place.

### Baseline self-assessment

Once enrolled, each participant was provided with a self-assessment kit to obtain baseline measurements. This kit included a pre-programmed accelerometer to be worn for at least seven consecutive days from morning to evening, and self-administered questionnaires (International Physical Activity Questionnaire (IPAQ), and Short Form 36 (SF-36)). The IPAQ questionnaire was chosen because it is validated for use in primary care and because it assesses the amount of physical activity the patient reports to provide the equivalent Metabolic Equivalent Task-minutes (MET-min) [23]. The accelerometer provided no feedback to participants on their daily activity. Once completed, the participants returned the self-assessment kit by post and data treatment was centralised. The GPs and participants did not receive baseline results before the end of the study.

### Randomisation

After this initial self-assessment period, the participants returned for the first study visit with their GP during which they were randomised to either the verbal advice (control) or PPIL (Prescription, Pedometer, Information, Logbook) group. Participants were randomised on a 1:1 allocation ratio using an internet-based dynamic randomisation software (details are available in the published protocol [23]). The randomisation algorithm was based on nine criteria: diabetes (yes/no), body mass index (BMI) (< 30, ≥ 30), sex (female/male), hypertension (yes/no), employment status (employed/unemployed), hypercholesterolaemia (yes/no), age (35–54 years, 55–74 years), education level (≤ high school diploma, > high school diploma), and follow-up doctor. The Methodology and Biostatistics Department of Angers University Hospital performed the concealed and centralised randomisation.

### Intervention delivery

In the verbal advice group, the GP verbally advised patients to increase their daily physical activity using the following standardised wording: “Try to do at least 15 min of brisk walking or another activity that makes you breathe faster than normal every day of the week”. The GP repeated this advice every three months during scheduled follow-up consultations if the patient was still judged as being insufficiently active.

In the PPIL group, the GP wrote a personalised physical activity prescription stating the number of daily steps to be taken and provided a pedometer (Omron HJ-321-E, a compact tri-axial pedometer to be carried in a pocket or a bag), information about the benefits of physical activity, and a logbook to keep daily physical activity records. The GP and participant agreed on an achievable target number of steps to be taken each day above the participant’s baseline activity level. This was then indicated on the written prescription. For example, “4000 steps above your usual number of steps”. The GP encouraged the participant to try reaching the prescribed target over a three-month period by setting intermediate weekly goals.

The first week, participants continued their normal daily routine and recorded their average daily step count. The second week, the intermediate weekly goal was set by adding a mutually agreed number of steps to their normal average step count. This continued until the target was reached. Activities not logged by the pedometer such as swimming, cycling, and gardening, could be recorded in the logbook using the included conversion table. Participants were asked to bring their logbook to each follow-up consultation at study month 3, 6, and 9 during which the participant and GP reassessed the target step number together.

At study month 3 and 12, participants received the self-assessment kit containing the accelerometer to wear for seven consecutive days and the self-administered questionnaires (IPAQ, SF-36, and Determinants of Physical Activity Questionnaire (DPAQ)). After seven days, they returned the kits for centralised treatment.

### Outcomes

The primary outcome was the change in total weekly energy expenditure at 3 months compared with the baseline, measured using an accelerometer, in MET-min. Secondary outcomes for objective measures were changes at 3 and 12 months compared with baseline for accelerometer-recorded step count and time spent performing light, moderate and vigorous physical activity, weight, waist circumference, and blood pressure.

Secondary outcomes for self-reported measures were changes at 3 and 12 months compared with baseline for weekly physical activity level in MET-min calculated using IPAQ and quality of life measured using the SF-36 questionnaire, and perceived barriers to physical activity at 3 and 12 months assessed using the DPAQ.

### Data collection

Prior to randomisation, sociodemographic data were collected including age, gender, education level, family unit (living alone or with family), socio-professional category, occupation, employment type, living environment and distance to workplace. Data concerning tobacco use, hypercholesterolaemia, hypertension, diabetes, medical history, and drug history were also collected.

At baseline, 3 and 12 months participants recorded energy expenditure, physical activity intensity, and step counts over a consecutive 7-day period using the self-administered IPAQ and the Actigraph wGT3X-BT accelerometer which was attached to an elastic belt and worn around the waist from morning to bedtime [24]. They also recorded quality of life using the SF-36 self-administered questionnaire at the same time points [25]. At 3 and 12 months, perceived barriers to behavioural change (barriers to physical activity) were assessed using the self-administered DPAQ [18].

During the follow-up consultations, the GP recorded blood pressure, weight, and waist circumference using their own equipment. The GP also recorded adherence wearing the pedometer (PPIL group), changes in drug treatment, possible difficulties, and adverse events.

### Accelerometer data processing

Data were processed using ActiLife5 software. The produced report contained daily information about wear time, number of steps, METs, and time spent doing activities of different intensity levels. Daily data was only valid if the accelerometer wear time had been at least 600 min for that day. If wear time for a certain day was less, or the accelerometer was not worn at all, imputation technique was applied, replacing invalid day data with the previous valid day.

Actigraph counts were sampled over 60-s periods (epochs). Per-epoch activity intensity was coded using integrated Freedson adult cut-point ranges (specifically, sedentary = 0–99 counts, light = 100–1951 counts, moderate = 1952–5724 counts, vigorous = 5725–9498 counts, and very vigorous activity > 9498 counts). To obtain weekly time spent doing a given activity, 7-day data were summarised.

METs for each day were obtained using the integrated Swartz Adult Overground & Lifestyle MET rate algorithm. Daily minutes when the accelerometer was not worn (1440 min – wear time) were rated at 1 MET. Daily MET-min was calculated as [METs x wear time + (1440 min—wear time)]. To obtain weekly energy expenditure (MET-min/week), 7-day data were summarised.

### IPAQ data processing

IPAQ contains questions on vigorous activity, moderate activity, and walking. To calculate MET-min/week, conventional MET values (walking = 3.3, moderate activity = 4, vigorous activity = 8) were multiplied by minutes spent doing the activity and again by the number of days that activity was performed. For example, if a participant reported walking for 30 min seven days a week, the MET-min for that activity are: 3.3 × 30 × 7 = 693 MET-min/week. Total MET-min/week for all activities is obtained by adding together the MET-min achieved in each category. Remaining minutes in the week were rated at 1 MET.

### Statistical and subgroup analyses

The sample size determination has been previously published [23]. Based on available data, 70 patients per group (140 total) were estimated to be sufficient to detect a between-group difference in total energy expenditure at 3 months of 105 MET-min/week (about 35 min of moderate activity per week), with a statistical power of 90%, an alpha risk of 5% and an estimate standard deviation of 185 MET-min/week.

Quantitative variables are expressed as mean (standard deviation). Qualitative variables are expressed as number and percentage. The study group characteristics before the intervention were compared using unpaired t-test (quantitative) and Fisher’s exact test (qualitative). The overall effect of physical activity prescription was compared between the verbal and prescription groups using linear mixed-effects models, to correlate observations on an individual level (different time points) and a centre level (different patients in the same medical centre) over three time points (0, 3 and 12 months). The effect of providing a written prescription for physical activity is the group and time interaction coefficient showing the variation between groups for a one-month increase in time. This physical therapy prescription effect will be expressed as a coefficient with 95% CI. This method follows the De Livera, Zaloumis and Simpson recommendations [26]. The effect on the whole period was obtained by multiplying this coefficient by 12. All observations with at least one post-randomisation measurement for two periods (3 and 12 months) were included in the analysis. For these models, p-values were calculated using Satterthwaite’s degrees of freedom method. This method is appropriated and commonly used for the analysis of repeated outcome measures. Adjusted *P* value ≤ 0.05 was considered significant. All fixed-effect coefficients can be found in supplementary tables 1 and 2.

Analyses were performed using Prism GraphPad 8.0.1 and R 4.2.1. with lmerTest package.

### Post-hoc analysis

All participants were expected to be insufficiently active at inclusion, based on self-reported activity. However, both the accelerometer and IPAQ results revealed heterogeneous participant baseline activity. To take these differences into account, a post-hoc subgroup analysis was performed among “initially active” and “initially inactive” subgroups between the verbal advice group and PPIL group. “Initially inactive” participants were defined as those recording less than 5000 steps/day at baseline as measured by the accelerometer. Energy expenditure (MET-min/week), number of steps/week and time spent doing moderate intensity activity (min/week) were compared between the “initially inactive” subgroups in the PPIL and verbal advice groups and between the “initially inactive” subgroups in each group using linear mixed effect models as previously described. An increase of more than 500 steps/week will be considered as clinically significant.

## Ethics approval

All methods were performed in accordance with the relevant guidelines and regulations. The protocol, information letter and informed consent certificate were approved by the Angers ethics committee and the French Health Products Safety Agency (study number 2014-A00332-45The French Data Protection Agency authorised the collection of identifying data (CCTIRS/CNIL approval number 14.559). The PEPPER trial is registered in the US National Institutes of Health Clinical Trials Registry under number NCT02317003 (15/12/2014). The study was designed and reported in accordance with the CONSORT (Consolidated Standards of Reporting Trial) statement [27].

## Results

### Study population

In total, 125 participants were recruited, 121 of which were randomised being 86% of the planned sample size (*n* = 140). Recruitment was stopped at this point as it was not possible to recruit more individuals within a reasonable time and recruiting 15 more individuals would add only minor modifications to the results.

**Fig. 1:**
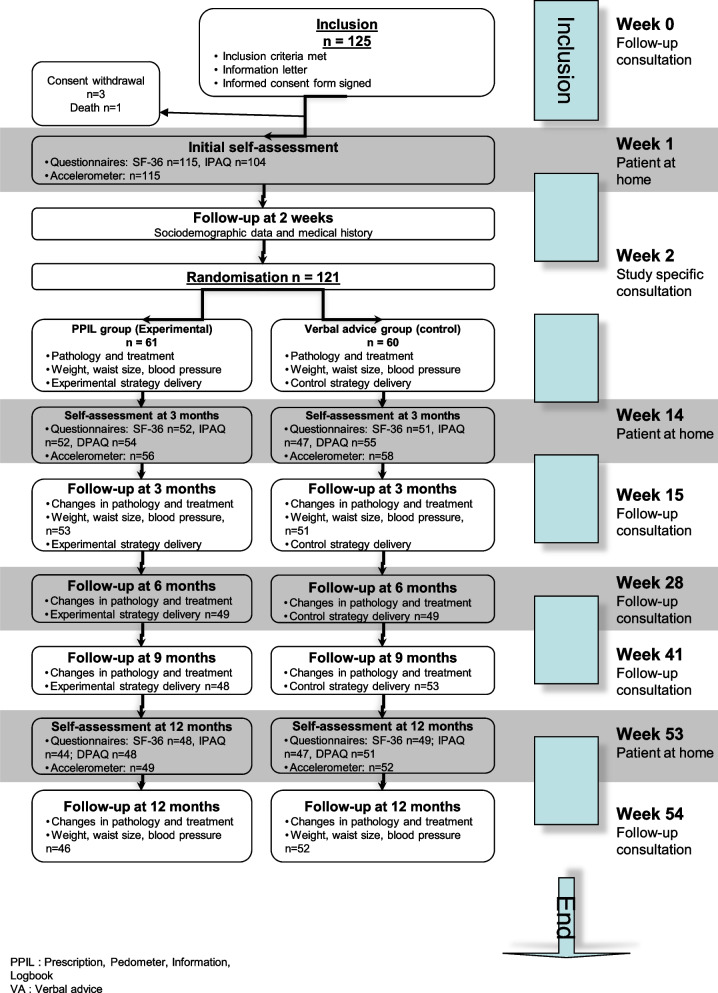
Recruitment and follow-up flowchart

Sixty-one participants were randomised into the PPIL group and 60 into the verbal advice group (Fig. 1). For the main analysis, all participants with at least one post-randomisation time-point (T = 3 or T = 12) were included.

### Baseline biomedical characteristics and sociodemographic data

Baseline sociodemographic and biomedical characteristics are detailed in Table 1.

**Table 1:** Baseline sociodemographic and biomedical characteristics of both groups

	**Verbal advice Group** **(*n* = 60)**	**PPIL Group** **(*n* = 61)**
Gender – Male, n (%)	35 (58)	31 (51)
Age in years, mean (SD)	60 (9)	59 (8)
BMI in Kg/m^2^, mean (SD)	30 (4)	31 (5)
**Cardiovascular risk factors**		
Diabetes, n (%)	25 (42)	24 (39)
Arterial hypertension, n (%)	50 (83)	47 (77)
Hypercholesterolaemia, n (%)	24 (40)	28 (46)
Smoker, n (%)	7 (12)	9 (15)
**Sociodemographic data**		
Education level ≤ high school diploma, n (%)	41 (68)	41 (67)
Living alone, n (%)	13 (22)	12 (20)
Living environment, n (%)		
- urban,	7 (12)	9 (15)
- semi-urban,	33 (55)	28 (46)
- rural	20 (33)	24 (39)
Employed, n (%)	25 (42)	23 (38)

*PPIL* Prescription, Pedometer, Information, Logbook (Experimental group)

Men accounted for 51% of participants in the PPIL group and 58% in the verbal advice group. The education level for most patients in both groups did not exceed a high school diploma. Participants were mainly retired or unemployed and living with their partner in semi-urban or rural areas. Employed participants, worked at least 10 km from their home, and occupations were mainly sedentary.

The mean BMI was similar in both groups and around 50% of participants in both groups were obese. Adherence to study procedures in the PPIL group was good. Only 4 out of 53 participants at month 3 (8%), 11 out of 49 patients at month 6 (22%), and 10 out of 48 patients at month 9 (21%) reported non-regular pedometer and logbook use.

### Baseline activity

Baseline activity levels for all included participants were heterogeneous, with weekly steps ranging from 13,600 to 98,700 steps, time spent doing moderate-intensity activity ranging from 1 to 625 min/week, and energy expenditure ranging from 11,940 to 20,540 MET-min/week.

### Physical activity

For the primary outcome of change in weekly energy expenditure at 3 months compared with baseline, no statistically significant between-group difference was observed. However, a clinically increase in time spent doing moderate-intensity activity was observed, which reached 48 min after 12 months (95% CI: 8 – 89). This increased activity intensity was also associated with a higher number of steps, 5256 steps/week increase (95% CI: 660 – 9852) in the PPIL group compared with the verbal advice group (Table 3).

#### Weight, waist circumference and blood pressure

Body weight, waist circumference and blood pressure did not significantly change in either group (Tables 2 and 3).

**Table 2:** Physical activity indicators at baseline, 3 and 12 months in the verbal advice and PPIL groups

Variable	Verbal advice group						PPIL group					
	**Baseline**		**M3**		**M12**		**Baseline**		**M3**		**M12**	
	**Mean (SD)**	**n**	**Mean (SD)**	**n**	**Mean (SD)**	**n**	**Mean (SD)**	**n**	**Mean (SD)**	**n**	**Mean (SD)**	**n**
Body weight, kg	85 (14)	60	86 (14)	51	85 (15)	52	87 (16)	61	87 (17)	53	85 (18)	46
Waist circumference, cm	106 (11)	60	106 (12)	51	107 (19)	50	106 (12)	59	106 (13)	52	108 (21)	44
Systolic blood pressure, mmHg	135 (11)	60	137 (12)	51	135 (11)	52	132 (12)	61	133 (11)	53	134 (12)	46
Diastolic blood pressure, mmHg	76 (7)	60	76 (9)	51	74 (9)	52	75 (10)	61	74 (11)	53	74 (10)	46
Accelerometer wear time, min/day	808 (82)	59	783 (89)	58	796 (97)	52	811 (93)	56	815 (101)	56	808 (92)	49
Energy expenditure, MET-min/week (accelerometer)	15,082 (1645)	59	14,755 (1626)	58	14,821 (1618)	52	14,811 (1540)	56	14,791 (1603)	56	14,847 (1653)	49
Steps number per week (accelerometer)	48,414 (18,818)	59	44,784 (19,317)	58	46,768 (20,011)	52	44,102 (16,850)	56	46,534 (16,924)	56	47,163 (18,749)	49
Time spent at light activity, min/week (accelerometer)	2195 (655)	59	2048 (623)	58	2164 (647)	52	2100 (567)	56	2070 (607)	56	2087 (645)	49
Time spent at moderate activity, min/week (accelerometer)	172 (122)	59	163 (129)	58	145 (101)	52	154 (134)	56	172 (134)	56	172 (131)	49
Time spent at vigorous activity, min/week (accelerometer)	2 (7)	59	2 (10)	58	5 (19)	52	0 (2)	56	0 (0)	56	0 (1)*	49
SF-36 Physical component, 0-to-100 scale	48 (8)	58	50 (8)	51	49 (7)	49	46 (8)	57	47 (8)	52	48 (8)	48
SF-36 Mental component, 0-to-100 scale	50 (9)	58	50 (10)	51	51 (8)	49	45 (11)	57	48 (9)	52	46 (11)	48
DPAQ global score, 1-to-4 scale			2.83 (0.39)	55	2.89 (0.4)	51			2.83 (0.43)	55	2.83 (0.47)	49
IPAQ Energy expenditure, MET-min/week	13,395 (3985)	54	12,963 (2604)	46	12,870 (2772)	47	12,372 (3490)	50	12,959 (2989)	51	12,717 (2797)	44
IPAQ moderate activity, min/week	257 (495)	59	230 (451)	57	213 (303)	50	223 (522)	60	204 (357)	57	254 (448)	50
IPAQ vigorous activity, min/week	165 (329)	59	122 (202)	55	150 (248)	51	83 (189)	60	159 (275)	56	108 (242)	49
IPAQ walking time, min/week	646 (936)	44	516 (587)	39	473 (475)	42	431 (713)	40	457 (409)	47	425 (368)	42

*PPIL* Prescription, Pedometer, Information, Logbook (Experimental group), *M3* Month 3, *M12* Month 12; *p < 0.05 vs. Control group

**Table 3:** Physical activity prescription effect between PPIL and verbal advice group

Model	Standardized mean effect for a4-weeks unit of time (95% CI)	*p*-value	Number of observations
Body weight, Kg	0 (-0.1 – 0.1)	0.787	109
Waist circumference, cm	0.1 (-0.3 – 0.5)	0.487	108
Systolic blood pressure, mmHg	0.3 (-0.1 – 0.7)	0.197	109
Diastolic blood pressure, mmHg	0.19 (-0.2 – 0.4)	0.486	109
Accelerometer wear time, min/day	0.8 (-15.8 – 17.5)	0.923	115
Energy expenditure MET-min/week (accelerometer)	17.9 (-16.2 – 52)	0.304	115
Number of steps/week (accelerometer)	438.4 (55.1 – 820.7)	0.026	115
Time spent doing light intensity activity, min/week (accelerometer)	0.2 (-14.8 – 15.2)	0.974	115
Time spent doing moderate intensity activity, min/week (accelerometer)	4 (0.7 – 7.4)	0.018	115
Time spent doing vigorous intensity activity, min/week (accelerometer)	-0.3 (-0.5 – 0)	0.093	115
SF-36 physical component, 0 to 100 scale	0.1 (-0.1 – 0.3)	0.29	110
SF-36 mental component, 0 to 100 scale	-0.2 (-0.5 – 0.1)	0.114	110
IPAQ energy expenditure, MET-min/week	38.7 (-87.4 – 165.5)	0.55	108
IPAQ moderate intensity activity min/week	7.4 (-10.1 – 24.9)	0.411	108
IPAQ vigorous intensity activity, min/week	0.6 (-11 – 12.3)	0.915	108
IPAQ walking time, min/week	8.8 (-16.6 – 34.4)	0.499	

#### IPAQ results

Results are presented in Tables 2 and 3. Weekly energy expenditure, time spent doing moderate and vigorous-intensity activity, and walking time as calculated from the IPAQ did not vary significantly over the timepoints or between groups.

When compared with the accelerometer results, the IPAQ results for weekly energy expenditure were 10–15% lower, weekly time spent doing moderate-intensity activity were around 40% greater with a three times greater standard deviation. Additionally, vigorous-intensity activity was reported by the IPAQ, but nearly undetected by the accelerometer.

#### Quality of life (SF-36)

No statistically significant difference was observed over time and between the groups in the SF-36 physical and mental components (Tables 2 and 3).

#### Barriers to physical activity (DPAQ)

Global DPAQ questionnaire scores are presented in Table 2 and the DPAQ panel results are detailed in Table 4. Only a moderate level of barriers was perceived (approximately 2.8 on a 1-to-4 scale with 1 being maximal barriers, and 4 being no barriers perceived). This did not vary over time or between groups.

**Table 4:** DPAQ panels at months 3 and 12

DPAQ panels	Verbal advice group				PPIL group			
	**M3**		**M12**		**M3**		**M12**	
	**Mean (SD)**	**n**		**n**		**n**		**n**
Knowledge	2.81 (0.79)	55	3.02 (0.68)	51	3.00 (0.81)	54	2.92 (70)	48
Environmental context & resources	3.13 (0.73)	55	3.06 (0.66)	51	2.93 (0.65)	55	3.08 (0.63)	48
Motivation & goals	2.82 (0.71)	55	2.82 (0.66)	51	2.92 (0.68)	53	2.87 (0.65)	49
Beliefs about capabilities	3.15 (0.80)	54	3.32 (0.66)	51	3.02 (0.82)	55	3.11 (0.80)	47
Skills	2.50 (0.65)	55	2.51 (0.64)	51	2.44 (0.69)	55	2.47 (0.72)	49
Emotion	3.25 (0.70)	55	3.39 (0.60)	51	3.13 (0.85)	54	3.25 (0.77)	47
Social influences	2.73 (0.83)	55	2.89 (0.77)	51	2.79 (0.79)	54	2.87 (0.55)	48
Beliefs about consequences	3.30 (0.52)	53	3.33 (0.52)	49	3.43 (0.53)	53	3.31 (0.79)	47
Action planning	2.45 (0.70)	53	2.48 (0.80)	50	2.52 (0.73)	53	2.43 (0.79)	47
Coping planning	2.37 (0.61)	52	2.41 (0.56)	49	2.46 (0.51)	52	2.45 (0.50)	45
Goal conflict	2.59 (0.50)	52	2.46 (0.68)	49	2.58 (0.54)	52	2.66 (0.54)	46

1-to-4 scale where 1 = maximal barriers, 4 = no barriers; *PPIL* Prescription, Pedometer, Information, Logbook, *M3* Month 3; *M12* Month 12

#### Post-hoc subgroup analysis: “initially inactive” subgroup

At baseline, 16/59 (27%) of the verbal advice participants and 17/56 (30%) of the PPIL participants had step counts lower than 35,000/week. Among these initially inactive people in the PPIL group, a slightly increase of 739 steps/week was observed compared to the initially inactive verbal subgroup (p-0.004) for a 4-weeks unit of time. Interestingly, the step count remained similar between both initially active subgroups (Table 5).

**Table 5:** Effect of physical activity prescription among initially inactive patients between the prescription and verbal advice group

Model	Standardized mean effect for a 4-weeks unit of time (95% CI)	*p*-value	Number of observations
Energy expenditure, MET-min/week (accelerometer)	34.8 (-13 – 82.4)	0.159	33
Number of steps/week (accelerometer)	739.4 (248.5 – 1224.5)	0.004	33
Time spent doing moderate-intensity activity, min/week (accelerometer)	3.3 (-0.7 – 7.4)	0.11	33

#### Difficulties reported by participants

Few difficulties were reported (Table 6).

**Table 6:** Difficulties reported by participants during the study

	**Verbal advice group**			**PPIL group**		
**Difficulty reported, number reporting each difficulty**	**Month 3 (*n* = 51)**	**Month 6 (*n* = 49)**	**Month 9 (*n* = 53)**	**Month 3 (*n* = 53)**	**Month 6 (*n* = 49)**	**Month 9 (*n *= 48)**
Difficulty using pedometer	0	0	0	9	4	3
Difficulty using the logbook	0	0	0	1	1	1
Leg/back pain	2		4	5	4	4
Motivation loss	6	6	4	3	8	6
Difficulty organising activity	2	6	4	1	5	8
Difficulty increasing activity	1	0	0	1	0	2
Difficulty maintaining activity	0	2	1	0	0	0

*PPIL* Prescription, Pedometer, Information, Logbook (Experimental group)

### Adverse events

There were five adverse events declared. In the PPIL group, one person had a leg trauma, one had Achilles tendinitis, and one had severe lumbago. In the verbal advice group, one was hospitalised for urogenital reasons. One patient died before randomisation. Adverse events were not related to the intervention.

### Medical treatments

Medical treatments remained globally unchanged throughout the study. However, in the verbal advice group, treatment dose was reduced at month 3 for one participant with hypertension, and at month 12 for one participant with diabetes and one with hypertension. In the PPIL group, treatment dose was reduced for one participant with diabetes, one with hypertension and one with hypercholesterolaemia.

## Discussion

### Main results

During the twelve-month study period, the written personalised prescription combined with a pedometer did not significantly modify total weekly energy expenditure. However, there was a sustained increase in weekly step count and weekly moderate-intensity activity duration in the PPIL group compared with the verbal advice group. The prescription appeared most effective in sedentary patients. Quality of life (SF-36), blood pressure, weight, and waist circumference were not substantially modified by either strategy. The DPAQ questionnaire revealed that participants perceived few barriers to physical activity. Participants adhered well to the intervention with around 80% of PPIL participants regularly using their pedometers and logbooks.

The reason that the effectiveness of the PPIL strategy was not clearly demonstrated in terms of weekly energy expenditure compared with the control could be due to the chosen accelerometer. This is because the cut-off ranges were developed for active people and not specifically for the primary care setting (Freedson adult cut-points for activity intensity and Swartz MET rate conversion for energy expenditure). It is therefore difficult to interpret the accelerometer report in our insufficiently active population at risk of cardiovascular disease. It is possible that improvements in activity could have been undetected, as accelerometer counts might not have reached the cut-off to be classified in the higher category. This is supported by a 40% higher averaged self-estimated moderate-intensity activity duration than that measured by the accelerometer, and self-reports of vigorous activity that was not detected on the accelerometer. Further research is required to calibrate accelerometer cut-off ranges specifically adapted for activity levels and energy expenditure in physically inactive older adults.

Furthermore, it is possible that any improvements in activity were “diluted” in the 24-h energy expenditure results because including all activities, even 1-MET activities, may have caused classification and/or confusion bias.

Overall, baseline activity levels were higher than expected in these insufficiently active participants. Although it is possible that wearing the accelerometer may have been a novelty for the participants and created a Hawthorne effect, which leads people to being more active when participating in research compared with daily life [28]. This also means values could be biased (artificially high), and possibly explains the unexpected trend for accelerometer readings to decrease in the verbal advice group at months 3 and 12.

Furthermore, baseline activity levels were heterogeneous which could have influenced obtained results, making them more dispersed and less accurate. Baseline measurements revealed that some participants needed to improve their activity more than others, who were much more active. This meant the groups were not perfectly equilibrated in terms of activity, explaining why the ratio of “initially inactive” participants appeared larger in the PPIL group. It may be that definitive inclusion based on initial assessment results would obtain a more homogeneous population; however, the GPs did not have the baseline estimation results at this time. GPs also did not have the estimation results at randomisation so discrepancies in initial activity were not considered.

We obtained only modest improvements for some, but not all the study outcomes. These efficacy data are consistent with the results from a recent Cochrane meta-analysis of 14 interventional studies including 4762 participants comparing the use of a pedometer with verbal advice on physical activity. Despite the heterogenous study quality in terms of control groups and strong attrition bias, no long-term change in participant physical activity levels or significant change in blood pressure was observed. However, as with our study, sedentary patients had a greater increase in physical activity, which was associated with improved quality of life (SF-36 mental component) [29]. This also concurs with recent trials in primary care using a pedometer-based green prescription which revealed consistent results with improved physical activity levels for specific subgroups (older and inactive patients), cost-effectiveness and good patient acceptability [30, 31]. Other strategies include the Swedish model which consists of patient-centred dialogue, personalised physical activity prescription and follow-up. This model has been shown to increase the level of physical activity in insufficiently active adults. It has been suggested that physical activity prescription models which follow the Swedish model could be implemented in routine healthcare to increase physical activity levels. However, it remains unclear which components of the Swedish model have the greatest effect on physical activity levels and whether all three components are really needed [32].

The fact that our results reveal modest improvements could still prove beneficial as evidenced in a 2019 meta-analysis which revealed that any physical activity, regardless of intensity, and less time being sedentary reduce mortality with a non-linear dose–response. In this study, the participants were divided into quartiles according to their physical activity level at inclusion and assessed using an accelerometer. After a median of 5.8 years follow-up, the hazard ratios for mortality were 1 for the reference group (sedentary), 0.48 (95% CI [0.43–0.54]) for the second quartile (lightly active), 0.34 (95% CI [0.26–0.45]) for the third quartile (moderately active) and 0.27 (95% CI [0.23–0.32]) for the fourth quartile (very active) [1].

In the present study, the written, personalised physical activity prescription was employed as a practical prevention tool for use in general practice to reduce cardiovascular risk. However, it is difficult to say whether prescribing physical activity is a primary or secondary prevention tool or whether it is both [31, 33, 34] since primary prevention aims to prevent a disease before it occurs, and secondary prevention aims to reduce the impact of a disease that has already occurred.

Participants and their GPs generally appeared motivated to participate in this study. Those enrolled in the verbal advice group were as motivated to participate in the study as those in the PPIL group. They undoubtedly appreciated that their GP recruited them in a study aiming to improve their quality of life and reduce their cardiovascular risk factors. This may explain why there was little difference between the groups, along with the possibility that the regular follow-ups made the control group perform better. Furthermore, participants adhered well to wearing the pedometer and completing the logbook, demonstrating this type of intervention is feasible in primary care. As an alternative to the pedometer, a smartphone app or connected watch could be used to self-track activity [35, 36].

Within a multidimensional treatment approach to reduce cardiovascular risk factors, the patient’s lifestyle is more than behaviours involving eating, physical activity, and substance use [37]. A holistic, patient-centred approach is required enabling personalised therapy, improved morbidity indicators and quality of life, and reduced healthcare costs [38, 39]. This means that to ensure physical activity prescriptions are patient-centred, the patient’s disease or disorder severity, functional capacities and motivation must be considered [40].

### Strengths and limitations

A strength of this randomised-controlled study is that the intervention was easy to apply, did not add additional workload on the GP or change usual practice, patient follow-up. Also, the resources, staff, financial input, and time spent were limited. Nevertheless, this pragmatic study conducted in a primary care setting with GP investigators suggests the results are possibly transposable to regular care.

In addition to the limitations inherent to this study design, other limitations existed. Blinding was not possible in the study intervention or anthropometric data collection due to the nature of these interventions. We observed a selection bias, because patients were more active than expected at baseline, and a Hawthorne effect is possible due to the non-blinded design. It was almost impossible to ensure that participants did not meet one another and discuss the study so there is the possibility that this may have occurred. It is also conceivable that patients in the control group decided to use their own pedometer thus potentially altering their physical activity levels. Furthermore, some patients may not have worn the pedometer correctly which could have minimised the results. Multiple comparisons could have enhanced alpha risk inflation. We also could assume a lack of statistical power due to the low number of included patients in the study.

### Future perspectives

Given the sample size and the study design, our findings could theoretically be generalised to a larger-scale population of insufficiently active or sedentary older adult patients in primary care. However further research is needed to properly assess the generalisability.

## Conclusion

Prescribing a personalised number of steps to inactive patients with cardiovascular risk factors in primary care did not significantly modify total weekly energy expenditure, but slightly increased the step count and moderate activity duration. Thus, prescribing personalised physical activity goals could therefore be an effective solution to encourage sedentary patients to engage in physical activity.

## Supplementary Information

**Additional file 1:** **SupplementaryTable 1. **Coefficients for overall mixed-model. 

**Additional file 2:** **Supplementary Table 2. **Coefficients for mixed-models.

## Data Availability

Datasets are available in request to the corresponding author; the raw data supporting the conclusions of this article will be made available by the authors, without undue reservation.

## Abbreviations

IPAQ: International Physical Activity Questionnaires
GP: General practitioner
DPAQ: Determinants of Physical Activity Questionnaire
SF-36: Short Form 36
PPIL: Prescription, Pedometer, Information, Logbook
VA: Verbal advice
CONSORT: Consolidated Standards of Reporting Trial
MET-min: Metabolic Equivalent Task-minute
