# Supplementary material for: Comparing physical activity prescription with verbal advice for general practice patients with cardiovascular risk factors: results from the PEPPER randomised controlled trial
Source: BMC Public Health. 2023 Jul 20;23:1402. doi: 10.1186/s12889-023-16302-6 (PMC10360325; doi:10.1186/s12889-023-16302-6)
Supplement: Supplementary file 1 — Additional file 1: SupplementaryTable 1. Coefficients for overall mixed-model. [file 12889_2023_16302_MOESM1_ESM.docx]

**Supplementary table 1 – Coefficients for overall mixed-model**

Coefficients interpretation: The coefficient time alone is interpreted as the effect of time independently of the group to which the patient belongs and the effect of the variable group alone is interpreted as a possible difference between the baseline groups. The interaction term is indeed the effect of the intervention on a time unit (a month).

| **Results** | **Modele** | **Estimate (95% CI)** | **P-value** | **Number of observations** |
| --- | --- | --- | --- | --- |
| (Intercept) | Body weight, kg | 84.9(81 - 88.9) | < 0.001 | 109 |
| groupPPIL | Body weight, kg | 1.7(-3.8 - 7.3) | 0.54 | 109 |
| time | Body weight, kg | 0(-0.1 - 0) | 0.356 | 109 |
| Difference in change for a monthly increase in time | Body weight, kg | 0(-0.1 - 0.1) | 0.787 | 109 |
| (Intercept) | Waist circumference, cm | 106(101.6 - 110.4) | < 0.001 | 108 |
| groupPPIL | Waist circumference, cm | -0.2(-5.1 - 4.8) | 0.937 | 108 |
| time | Waist circumference, cm | 0.1(-0.2 - 0.4) | 0.389 | 108 |
| Difference in change for a monthly increase in time | Waist circumference, cm | 0.1(-0.3 - 0.5) | 0.487 | 108 |
| (Intercept) | Systolic blood pressure, mmHg | 135.3(132.2 - 138.4) | < 0.001 | 109 |
| groupPPIL | Systolic blood pressure, mmHg | -3.4(-7 - 0.2) | 0.065 | 109 |
| time | Systolic blood pressure, mmHg | -0.1(-0.3 - 0.2) | 0.69 | 109 |
| Difference in change for a monthly increase in time | Systolic blood pressure, mmHg | 0.3(-0.1 - 0.7) | 0.197 | 109 |
| (Intercept) | Diastolic blood pressure, mmHg | 77.1(74.3 - 80) | < 0.001 | 109 |
| groupPPIL | Diastolic blood pressure, mmHg | -1.2(-3.9 - 1.5) | 0.386 | 109 |
| time | Diastolic blood pressure, mmHg | -0.1(-0.3 - 0.1) | 0.276 | 109 |
| Difference in change for a monthly increase in time | Diastolic blood pressure, mmHg | 0.1(-0.2 - 0.4) | 0.486 | 109 |
| (Intercept) | Accelerometer wear time, min/day | 5592.5(5404 - 5783.8) | < 0.001 | 115 |
| groupPPIL | Accelerometer wear time, min/day | 123.4(-98.8 - 344) | 0.276 | 115 |
| time | Accelerometer wear time, min/day | -6(-17.6 - 5.6) | 0.308 | 115 |
| Difference in change for a monthly increase in time | Accelerometer wear time, min/day | 0.8(-15.8 - 17.5) | 0.923 | 115 |
| (Intercept) | Energy expenditure, MET-min/week (accelerometer) | 14981.7(14559.5 - 15413.1) | < 0.001 | 115 |
| groupPPIL | Energy expenditure, MET-min/week (accelerometer) | -152.3(-712.6 - 404.1) | 0.594 | 115 |
| time | Energy expenditure, MET-min/week (accelerometer) | -13.5(-37.3 - 10.3) | 0.265 | 115 |
| Difference in change for a monthly increase in time | Energy expenditure, MET-min/week (accelerometer) | 17.9(-16.2 - 52) | 0.304 | 115 |
| (Intercept) | Steps number per week (accelerometer) | 47014.3(42088.3 - 51917.6) | < 0.001 | 115 |
| groupPPIL | Steps number per week (accelerometer) | -1897(-8456.9 - 4537.6) | 0.566 | 115 |
| time | Steps number per week (accelerometer) | -197.9(-464.1 - 69.5) | 0.148 | 115 |
| Difference in change for a monthly increase in time | Steps number per week (accelerometer) | 438.4(55.1 - 820.7) | 0.026 | 115 |
| (Intercept) | Time spent at light activity, min/week (accelerometer) | 2151.5(1983.6 - 2325.7) | < 0.001 | 115 |
| groupPPIL | Time spent at light activity, min/week (accelerometer) | -46.5(-261.5 - 169.1) | 0.673 | 115 |
| time | Time spent at light activity, min/week (accelerometer) | -0.4(-10.8 - 10.1) | 0.941 | 115 |
| Difference in change for a monthly increase in time | Time spent at light activity, min/week (accelerometer) | 0.2(-14.8 - 15.2) | 0.974 | 115 |
| (Intercept) | Time spent at moderate activity, min/week (accelerometer) | 171.6(142.1 - 201.8) | < 0.001 | 115 |
| groupPPIL | Time spent at moderate activity, min/week (accelerometer) | -11.1(-54.2 - 31.2) | 0.614 | 115 |
| time | Time spent at moderate activity, min/week (accelerometer) | -2.9(-5.2 - -0.6) | 0.016 | 115 |
| Difference in change for a monthly increase in time | Time spent at moderate activity, min/week (accelerometer) | 4(0.7 - 7.4) | 0.018 | 115 |
| (Intercept) | Time spent at vigorous activity, min/week (accelerometer) | 1.9(-0.2 - 4.1) | 0.094 | 115 |
| groupPPIL | Time spent at vigorous activity, min/week (accelerometer) | -1.6(-4.6 - 1.4) | 0.298 | 115 |
| time | Time spent at vigorous activity, min/week (accelerometer) | 0.2(0 - 0.4) | 0.022 | 115 |
| Difference in change for a monthly increase in time | Time spent at vigorous activity, min/week (accelerometer) | -0.3(-0.5 - 0) | 0.093 | 115 |
| (Intercept) | SF-36 Physical component, 0-to-100 scale | 48.3(46.1 - 50.4) | < 0.001 | 110 |
| groupPPIL | SF-36 Physical component, 0-to-100 scale | -2.4(-5.1 - 0.3) | 0.088 | 110 |
| time | SF-36 Physical component, 0-to-100 scale | 0(-0.1 - 0.2) | 0.656 | 110 |
| Difference in change for a monthly increase in time | SF-36 Physical component, 0-to-100 scale | 0.1(-0.1 - 0.3) | 0.29 | 110 |
| (Intercept) | SF-36 Mental component, 0-to-100 scale | 49(46.1 - 51.8) | < 0.001 | 110 |
| groupPPIL | SF-36 Mental component, 0-to-100 scale | -3.2(-6.6 - 0.1) | 0.058 | 110 |
| time | SF-36 Mental component, 0-to-100 scale | 0.1(-0.1 - 0.3) | 0.209 | 110 |
| Difference in change for a monthly increase in time | SF-36 Mental component, 0-to-100 scale | -0.2(-0.5 - 0.1) | 0.114 | 110 |
| (Intercept) | IPAQ Energy expenditure, MET-min/week | 13258.9(12511.3 - 14009.1) | < 0.001 | 108 |
| groupPPIL | IPAQ Energy expenditure, MET-min/week | -675.2(-1737.3 - 387.2) | 0.216 | 108 |
| time | IPAQ Energy expenditure, MET-min/week | -24.8(-113.1 - 62.6) | 0.58 | 108 |
| Difference in change for a monthly increase in time | IPAQ Energy expenditure, MET-min/week | 38.7(-87.4 - 165.5) | 0.55 | 108 |
| (Intercept) | IPAQ moderate activity, min/week | 288.2(172.3 - 408.8) | < 0.001 | 108 |
| groupPPIL | IPAQ moderate activity, min/week | -46.5(-200.2 - 113.5) | 0.557 | 108 |
| time | IPAQ moderate activity, min/week | -4(-16.2 - 8.1) | 0.521 | 108 |
| Difference in change for a monthly increase in time | IPAQ moderate activity, min/week | 7.4(-10.1 - 24.9) | 0.411 | 108 |
| (Intercept) | IPAQ vigorous activity, min/week | 168.4(105.2 - 231.6) | < 0.001 | 108 |
| groupPPIL | IPAQ vigorous activity, min/week | -39.2(-125.5 - 48) | 0.377 | 108 |
| time | IPAQ vigorous activity, min/week | -0.5(-8.5 - 7.5) | 0.897 | 108 |
| Difference in change for a monthly increase in time | IPAQ vigorous activity, min/week | 0.6(-11 - 12.3) | 0.915 | 108 |
| (Intercept) | IPAQ walking time, min/week | 622(463.2 - 780.8) | < 0.001 | 100 |
| groupPPIL | IPAQ walking time, min/week | -188.1(-411.6 - 35.3) | 0.102 | 100 |
| time | IPAQ walking time, min/week | -9.5(-27.5 - 8.4) | 0.301 | 100 |
| Difference in change for a monthly increase in time | IPAQ walking time, min/week | 8.8(-16.6 - 34.4) | 0.499 | 100 |
