# Supplementary material for: Comparing physical activity prescription with verbal advice for general practice patients with cardiovascular risk factors: results from the PEPPER randomised controlled trial
Source: BMC Public Health. 2023 Jul 20;23:1402. doi: 10.1186/s12889-023-16302-6 (PMC10360325; doi:10.1186/s12889-023-16302-6)
Supplement: Supplementary file 2 — Additional file 2: Supplementary Table 2. Coefficients for mixed-models. [file 12889_2023_16302_MOESM2_ESM.docx]

**Supplementary table 2 – Coefficients for mixed-models**

Coefficients interpretation: The coefficient time alone is interpreted as the effect of time independently of the group to which the patient belongs and the effect of the variable group alone is interpreted as a possible difference between the baseline groups. The interaction term is indeed the effect of the intervention on a time unit (a month).

**1 - Active patients model according to the IPAQ baseline results**

| **Results** | **Modele** | **Estimate (95% CI)** | **P-value** | **Number of observations** |
| --- | --- | --- | --- | --- |
| (Intercept) | Energy expenditure, MET-min/week (accelerometer) | 15463.4(14975 - 15953.6) | < 0.001 | 57 |
| groupPPIL | Energy expenditure, MET-min/week (accelerometer) | -551.5(-1337.2 - 234.1) | 0.175 | 57 |
| time | Energy expenditure, MET-min/week (accelerometer) | -22.4(-56.2 - 11.6) | 0.198 | 57 |
| Difference in change for a monthly increase in time | Energy expenditure, MET-min/week (accelerometer) | -8.1(-62.2 - 45.9) | 0.769 | 57 |
| (Intercept) | Steps number per week (accelerometer) | 52295.7(46136.9 - 58369.1) | < 0.001 | 57 |
| groupPPIL | Steps number per week (accelerometer) | -7006(-16771.7 - 2757.6) | 0.166 | 57 |
| time | Steps number per week (accelerometer) | -203.5(-593.8 - 189.5) | 0.311 | 57 |
| Difference in change for a monthly increase in time | Steps number per week (accelerometer) | 430.5(-195.8 - 1054.8) | 0.18 | 57 |
| (Intercept) | Time spent at moderate activity, min/week (accelerometer) | 200.9(160.7 - 241.2) | < 0.001 | 57 |
| groupPPIL | Time spent at moderate activity, min/week (accelerometer) | -35.5(-100.2 - 29.1) | 0.288 | 57 |
| time | Time spent at moderate activity, min/week (accelerometer) | -3.5(-6.8 - -0.1) | 0.044 | 57 |
| Difference in change for a monthly increase in time | Time spent at moderate activity, min/week (accelerometer) | 4.6(-0.7 - 9.9) | 0.093 | 57 |

**2 – Inactive patients model according to the IPAQ baseline results**

| **Results** | **Modele** | **Estimate (95% CI)** | **P-value** | **Number of observations** |
| --- | --- | --- | --- | --- |
| (Intercept) | Energy expenditure, MET-min/week (accelerometer) | 14299.1(13612.4 - 15007.6) | < 0.001 | 56 |
| groupPPIL | Energy expenditure, MET-min/week (accelerometer) | 486.1(-300.5 - 1278.9) | 0.231 | 56 |
| time | Energy expenditure, MET-min/week (accelerometer) | -0.6(-34.1 - 33) | 0.972 | 56 |
| Difference in change for a monthly increase in time | Energy expenditure, MET-min/week (accelerometer) | 27.4(-16.6 - 71.2) | 0.225 | 56 |
| (Intercept) | Steps number per week (accelerometer) | 40466.1(32648.3 - 48651.5) | < 0.001 | 56 |
| groupPPIL | Steps number per week (accelerometer) | 5282.8(-2999.2 - 13587) | 0.214 | 56 |
| time | Steps number per week (accelerometer) | -181.3(-550 - 188.7) | 0.338 | 56 |
| Difference in change for a monthly increase in time | Steps number per week (accelerometer) | 388.4(-96.1 - 870.8) | 0.118 | 56 |
| (Intercept) | Time spent at moderate activity, min/week (accelerometer) | 123.6(72.4 - 174.3) | < 0.001 | 56 |
| groupPPIL | Time spent at moderate activity, min/week (accelerometer) | 39.5(-19 - 95.6) | 0.177 | 56 |
| time | Time spent at moderate activity, min/week (accelerometer) | -1.7(-5 - 1.6) | 0.321 | 56 |
| Difference in change for a monthly increase in time | Time spent at moderate activity, min/week (accelerometer) | 2.8(-1.6 - 7.1) | 0.22 | 56 |

**3- Active patients model according to the baseline number of steps**

| **Results** | **Modele** | **Estimate (95% CI)** | **P-value** | **Number of observations** |
| --- | --- | --- | --- | --- |
| (Intercept) | Energy expenditure, MET-min/week (accelerometer) | 15571.4(15130 - 16013.1) | < 0.001 | 82 |
| groupPPIL | Energy expenditure, MET-min/week (accelerometer) | -51.3(-618 - 495.8) | 0.855 | 82 |
| time | Energy expenditure, MET-min/week (accelerometer) | -18.2(-47.8 - 11.4) | 0.231 | 82 |
| Difference in change for a monthly increase in time | Energy expenditure, MET-min/week (accelerometer) | 9.2(-34.3 - 53) | 0.679 | 82 |
| (Intercept) | Steps number per week (accelerometer) | 54113.6(49143.5 - 58938.5) | < 0.001 | 82 |
| groupPPIL | Steps number per week (accelerometer) | -1427.7(-7955.4 - 4835.3) | 0.657 | 82 |
| time | Steps number per week (accelerometer) | -164.1(-503.1 - 175.6) | 0.345 | 82 |
| Difference in change for a monthly increase in time | Steps number per week (accelerometer) | 305.5(-194.1 - 805.7) | 0.233 | 82 |
| (Intercept) | Time spent at moderate activity, min/week (accelerometer) | 209.4(171 - 247.1) | < 0.001 | 82 |
| groupPPIL | Time spent at moderate activity, min/week (accelerometer) | -7.4(-59.6 - 41.1) | 0.763 | 82 |
| time | Time spent at moderate activity, min/week (accelerometer) | -3.9(-6.8 - -0.9) | 0.011 | 82 |
| Difference in change for a monthly increase in time | Time spent at moderate activity, min/week (accelerometer) | 4.1(-0.3 - 8.4) | 0.069 | 82 |

**4- Inactive patients model according to the baseline number of steps**

| **Results** | **Modele** | **Estimate (95% CI)** | **P-value** | **Number of observations** |
| --- | --- | --- | --- | --- |
| (Intercept) | Energy expenditure, MET-min/week (accelerometer) | 13191.5(12640.1 - 13725.4) | < 0.001 | 33 |
| groupPPIL | Energy expenditure, MET-min/week (accelerometer) | 144.3(-504.2 - 755.5) | 0.649 | 33 |
| time | Energy expenditure, MET-min/week (accelerometer) | -0.8(-36 - 34.6) | 0.964 | 33 |
| Difference in change for a monthly increase in time | Energy expenditure, MET-min/week (accelerometer) | 34.8(-13 - 82.4) | 0.159 | 33 |
| (Intercept) | Steps number per week (accelerometer) | 26061.3(21433.2 - 30590.2) | < 0.001 | 33 |
| groupPPIL | Steps number per week (accelerometer) | 1616.6(-4636.3 - 7433) | 0.591 | 33 |
| time | Steps number per week (accelerometer) | -273(-631.1 - 91.2) | 0.143 | 33 |
| Difference in change for a monthly increase in time | Steps number per week (accelerometer) | 739.4(248.5 - 1224.5) | 0.004 | 33 |
| (Intercept) | Time spent at moderate activity, min/week (accelerometer) | 53.9(17 - 90.2) | 0.007 | 33 |
| groupPPIL | Time spent at moderate activity, min/week (accelerometer) | 13.3(-35.5 - 60.2) | 0.586 | 33 |
| time | Time spent at moderate activity, min/week (accelerometer) | 0(-3 - 3) | > 0.99 | 33 |
| Difference in change for a monthly increase in time | Time spent at moderate activity, min/week (accelerometer) | 3.3(-0.7 - 7.4) | 0.11 | 33 |
